# Postoperative Chemoradiotherapy versus Postoperative Chemotherapy for Completely Resected Gastric Cancer with D2 Lymphadenectomy: A Meta-Analysis

**DOI:** 10.1371/journal.pone.0068939

**Published:** 2013-07-18

**Authors:** Yuan-Yuan Huang, Qiong Yang, Si-Wei Zhou, Ying Wei, Yan-Xian Chen, De-Rong Xie, Bei Zhang

**Affiliations:** 1 Department of VIP, Sun Yat-sen University Cancer Center, Sun Yat-sen University, Guangzhou, P.R. China; 2 Department of Oncology, Sun Yat-sen Memorial Hospital, Sun Yat-sen University, Guangzhou, P.R. China; University of Wisconsin School of Medicine and Public Health, United States of America

## Abstract

**Background:**

Both chemoradiotherapy and chemotherapy are used in postoperative adjuvant therapy for resected gastric cancer. However, it is controversial whether chemoradiotherapy or chemotherapy is the optimal strategy for patients with gastric cancer after D2 lymphadenectomy. The present meta-analysis aims to provide more evidence on the relative benefits of adjuvant therapies in this setting.

**Methods:**

We conducted a systematic review of randomized controlled trials, extracted time-to-event data using Tierney methods (when not reported), and performed meta-analysis to obtain the relative hazards of adjuvant chemoradiotherapy to chemotherapy on efficacy and toxicities.

**Results:**

A total of 895 patients from 3 randomized controlled trials were identified for this meta-analysis. All patients were from Asian countries. Our results showed that postoperative chemoradiotherapy significantly improved locoregional recurrence-free survival [LRRFS: hazard ratio (HR) = 0.53, 95% CI = 0.32–0.87, p = 0.01] and disease-free survival (DFS: HR = 0.72, 95% CI = 0.59–0.89, p = 0.002); however, the improvement of distant metastasis recurrence-free survival (DMRFS: HR = 0.86; 95% CI = 0.66–1.11, p = 0.25) and overall survival (OS: HR = 0.79, 95% CI = 0.61–1.03, p = 0.08) were non-significant. The main grade 3 or 4 toxicities were equivalent between the two groups.

**Conclusion:**

In non-selected Asian patients with resected gastric cancer who underwent D2 lymphadenectomy, postoperative chemoradiotherapy improved LRRFS and DFS but might not improve OS compared to postoperative chemotherapy.

## Introduction

Globally, gastric cancer is the third leading cause of cancer related death among men and the fifth among women [1]. The primary curative treatment of gastric carcinoma is surgical resection [2]. Complete resection with adequate margins is widely considered as a standard goal, whereas the extent of lymph node dissection remains controversial. Irrespective of the surgical procedure used for treatment of gastric cancer, it is a consensus that patients with resected gastric cancer should receive adjuvant treatment.

In the last decade, postoperative chemoradiotherapy has become the preferred strategy for resected gastric cancer because the INT-0116 trial suggested that postoperative chemoradiotherapy had a survival advantage over observation. However, INT-0116 trial has been criticized for suboptimal surgery with 54% and 36% of patients receiving D0 and D1 dissections, respectively [3], [4]. Recently, gastrectomy with D2 lymphadenectomy has become the standard surgical procedure for curable gastric cancer in eastern Asia. It is also a recommended operation in European countries because of a reduction in gastric cancer-specific deaths with D2 dissection demonstrated in Dutch Gastric Cancer Group trial [5]. ACTS-GC and CLASSIC trials have shown that postoperative chemotherapy reduces risk of relapse and death in patients with gastric cancer after D2 lymphadenectomy [6], [7]. However, still, about 10% patients eventually have local relapse after D2 curative resection [6], [7]. Therefore, it is necessary to explore whether radiation added to adjuvant chemotherapy further improves survival for gastric cancer patients after D2 curative gastrectomy.

To our knowledge, there are three phase III randomized controlled trials (RCTs) which directly compared postoperative chemoradiotherapy with postoperative chemotherapy for patients with gastric cancer after D2 curative gastrectomy. Overall, two in three RCTs do not find any differences in overall survival (OS) and disease-free survival (DFS) between the two approaches [8], [9]. One RCT suggests that chemoradiotherapy improves DFS compared with chemotherapy [10]. Due to the inconsistent results, we attempted to explore this issue by meta-analysis.

## Methods

### Literature Search

A systematic review of eligible RCTs was performed by searching the electronic databases, which consist of Cochrane Central Register of Controlled Trials, PubMed, EMBASE, ISI Web of Knowledge, Chinese biomedical literature service system (SinoMed), ASCO abstracts, and ESMO abstracts. The keywords used for search were as follow: “gastric cancer”, “stomach neoplasms”, “chemoradiotherapy”, “chemoradiation”, “chemotherapy”, “D2”, and “combined modality therapy”. The search was limited to RCTs in English language. The deadline of this search was October 31, 2012. The reference lists of articles identified and relevant meta-analysis were searched manually to find other relevant articles. Meta-analysis was conducted according to the Preferred Reporting Items for Systematic Reviews and Meta-Analyses (PRISMA) statement [11], [12]. The PRISMA checklist was showed in Checklist S1.

### Trial Selection and Quality Assessment

All RCTs that compared chemoradiotherapy with chemotherapy in the setting of adjuvant therapy for resected gastric cancer with D2 lymphadenectomy were included in the present meta-analysis. If the same population appeared in other publications, the article that provided the most complete follow-up data on survival was selected. Methodological quality of the trials was assessed using a validated scale (range, 0 to 5) applied to items that influence intervention efficacy. The scale consists of items pertaining to randomization, masking, dropouts, and withdrawals, which is reported by Jadad *et al* [13]. A trial was regarded as high quality trial with high external and internal validities if it scored more than 3 points.

### Data Extraction and Analysis

Two primary reviewers (YYH and QY) assessed all abstracts that were identified from the above-mentioned sources. Both reviewers independently selected potentially eligible abstracts according to inclusion criteria. If one of the reviewers considered an abstract potential eligible, the full text of article was retrieved and reviewed in detail by both reviewers. Disagreements were resolved by consensus or by the third reviewer (BZ). Hazard ratio (HR) and 95% confidence interval (95% CI) for OS, DFS, locoregional recurrence-free survival (LRRFS) and distant metastasis recurrence-free survival (DMRFS) were requested. Where published, HR and 95% CI were extracted directly from the original article. Where HR and 95% CI were not reported, they were calculated from published summary statistics or survival curve using Tierney method [14]. The following variables were extracted from each trial if available: total numbers of patients, age, sex, ECOG performance status, primary tumor site, Lauren classification, tumor stage, treatment regimens, endpoints, median follow-up time, Jadad scale score, and toxicities.

### Statistical Analysis

The primary end points were OS, DFS, LRRFS, and DMRFS after randomization. The secondary end point was toxicity. Survival variables were defined as generic inverse variance data. We standardized the resulting treatment effect to obtain an effect size by HR. Toxicity variables were defined as dichotomous data. We standardized the outcome variable to obtain an effect size by Risk Ratio (RR). Crude HRs and RRs with 95% CIs were used to assess the survival benefit and risk of toxicities between chemoradiotherapy group and chemotherapy group, respectively. The significance of the pooled results was determined by the Z-test, and P<0.05 was considered as statistically significant.

Heterogeneity assumption was checked by a chi-square-based Q-test and also expressed as I^2^. A P-value of more than 0.10 for the Q-test and I^2^ of less than 50% indicated a lack of heterogeneity across the trials. If P-value of heterogeneity test was more than 0.1 and I^2^ was less than 50%, fixed effect model was performed and random effect model was used vice versa. However, due to the fixed effect model tended to underestimate standard errors of pooled estimates, random effect model was used for the quantitative pooling [15]. An estimate of the potential publication bias was carried out by funnel plot. An asymmetric plot suggested a possible publication bias. The funnel plot asymmetry was assessed by Egger’s test. P<0.05 was considered representative of statistically significant publication bias [16]. The statistical tests for our meta-analysis were performed with RevMan software (version 5.1, Cochrane) and STATA version 10.0 (Stata Corporation, College Station, TX).

## Results

### Trial Flow, Characteristics, and Quality Appraisal

**Figure 1:**
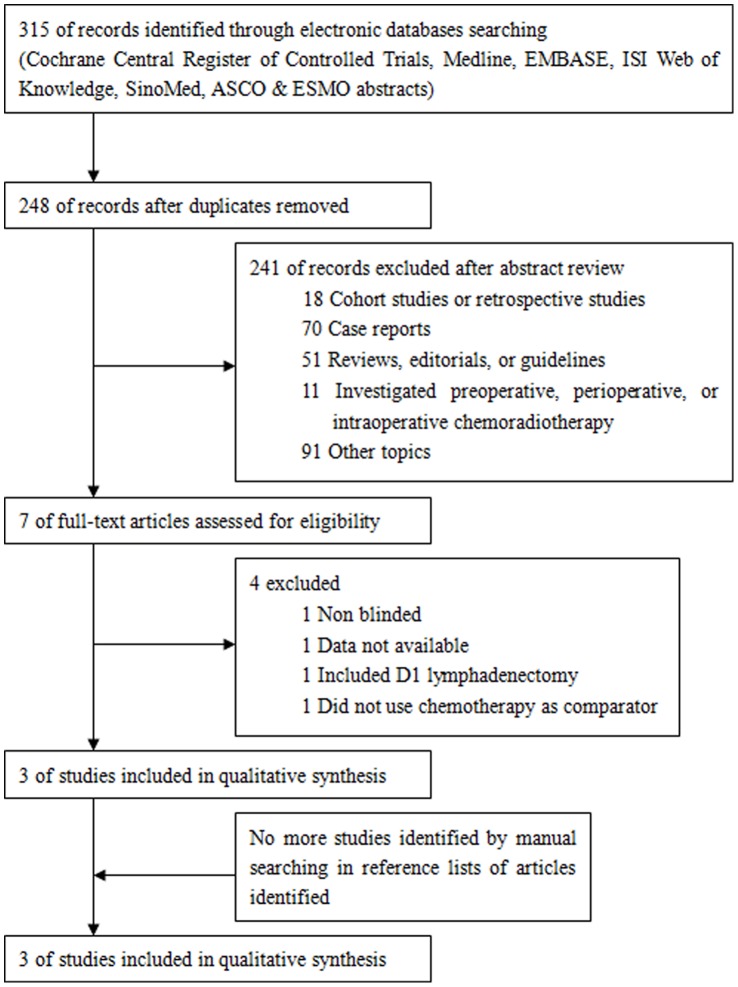
Flow chart of randomized controlled trials selection.

Figure 1 was the flow chart of RCTs selection for meta-analysis. A total of 895 patients from 3 RCTs were identified for this meta-analysis at last [8]–[10]. All patients were from Asian countries. Only one RCT uses intensive modulation radiotherapy (IMRT) as a part of concurrent chemoradiotherapy and shows DFS benefit from chemoradiotherapy [10]. One RCT uses capecitabine combined with cisplatin as chemotherapy regimen [8], the other two RCTs use the same chemotherapy regimen as that of INT-0116 [9], [10]. All RCTs don’t show that chemoradiotherapy has an OS advantage over chemotherapy. Table 1 and Table 2 showed important baseline characteristics and Jadad scores of selected trials.

**Table 1:** Characteristics of the selected RCTs.

References Characteristics	Lee et al 2012		Kim et al 2012		Zhu et al 2012	
	CT	CRT	CT	CRT	CT	CRT
Total number	228	230	44	46	165	186
Age year(range)	56(22–77)	56(28–76)	NR	NR	59(42–75)	56(38–73)
Sex No. (%)						
Male	153(67.1)	143(62.2)	25(56.8)	34(73.9)	126(76.4)	135(72.9)
Female	75(32.9)	87(37.8)	19(43.2)	12(26.1)	39(23.6)	51(27.1)
ECOG PS No. (%)						
0	96(42.1)	99(43.0)	27(61.4)	35(76.1)	NR	NR
1	132(57.9)	131(57.0)	17(38.6)	11(23.9)	NR	NR
Primary tumor site No.(%)						
Proximal	9(3.9)	13(5.7)	2(4.5)	3(6.5)	15(9.1)	30(16.1)
Body	112(49.1)	107(46.5)	19(43.2)	26(56.5)	33(20)	21(11.3)
Antrum	87(38.2)	90(39.1)	18(40.9)	14(30.5)	117(70.9)	135(72.6)
Multiple/diffuse	20(8.8)	20(8.7)	5(11.4)	3(6.5)	0	0
Lauren classification No. (%)						
Intestinal	88(38.6)	75(32.6)	15(34.1)	16(34.8)	NR	NR
Diffuse	130(57.0)	144(62.6)	24(54.5)	26(56.5)	NR	NR
Mixed	6(2.6)	8(3.5)	3(6.8)	2(4.3)	NR	NR
Not specified	4(1.8)	3(1.3)	2(4.5)	2(4.3)	NR	NR
Tumor stage No. (%)						
IB	50(21.9)	49(21.3)	0	0	15(9.1)	20(10.8)
II	86(37.7)	84(36.5)	0	0	30(18.2)	36(19.4)
III	65(28.6)	71(30.8)	31 (75)	34 (73.9)	96(58.2)	103(55.4)
IV(M0)	27(11.8)	26(11.3)	11(25)	12(26.1)	24(14.5)	27(14.5)
Treatment regimens	XP#	XP/XRT/XP*	FL<$>\vskip -1.5\scale 70%\raster="rg1"<$>	FL/RT§	FL<$>\vskip -1.5\scale 70%\raster="rg1"<$>	FL/IMRT¶
Endpoints	3-ys DFS: 74.2% vs 78.2%, p = 0.0862		5-ys DFS: 50.0% vs 60.9%, p = 0.246		5-ys RFS: 35.8% vs 45.2%, p = 0.029	
	OS not reached when data analyzed		5-ys OS: 54.6% vs 65.2%, p = 0.67		5-ys OS: 41.8% vs 48.4%, p = 0.122	
Follow-up month Median(range)	53.2(36.9–77.3)		86.7(60.3–116.5)		42.5	
Jadad scale score	3		3		3	

CT: chemotherapy, CRT: chemoradiotherapy, NR: not reported, 3-ys DFS: 3-year disease-free survival, 5-ys DFS: 5-year disease-free survival, 5-ys RFS: 5-year recurrence-free survival, 5-ys OS: 5-year overall survival.

# XP regimen: capecitabine 1000 mg/m2 twice daily on days 1 to 14; cisplatin 60 mg/m2 on day 1 every 3 weeks, totally 6 cycles.

* XP/XRT/XP: Two cycles of XP, then XRT (45 Gy of radiation at 1.8 Gy per day, 5 days per week, for 5 weeks with continuous capecitabine 825 mg/m2 twice daily during radiotherapy), followed by two cycles of XP.

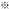
FL regimen: 5-fu 425 mg/m2, leucovorin 20 mg/m2, for 5 days with a 4-week interval, totally 5 cycles.

§ FL/RT: 1 cycle of FL, then RT(45 Gy of radiation at 1.8 Gy per day, 5 days per week, for 5 weeks with 2 cycles of FL), followed by two cycles of FL.

¶ FL/IMRT: 1 cycle of FL, then IMRT (45 Gy of radiation at 1.8 Gy per day, 5 days per week, for 5 weeks with 2 cycles of FL), followed by two cycles of FL.

**Table 2:** Main grade 3/4 toxicities of selected RCTs.

References	Regimens	N	Nausea/Vomit n (%)	Neutropenia n (%)	Anemia n (%)	Thrombocyto- penia n (%)
Lee et al	XP/XRT	230	35(15.4)	110(48.4)	1(0.4)	2(0.9)
	XP	228	36(15.9)	92(40.7)	4(1.7)	0(0)
Kim et al	FL/RT	41	*	*	*	*
	FL	45	*	*	*	*
Zhu et al	FL/IMRT	186	5(4.3)	14(7.5)	0(0)	0(0)
	FL	165	0(0)	12(7.3)	0(0)	0(0)

XP: capecitabine+cisplatin, XRT: radiotherapy with capecitabine, FL: fluorouracil plus leucovorin, RT: radiotherapy, IMRT: intensive modulation radiotherapy;

* Grade 3/4 hematologic and gastrointestinal toxicities occurred in 19.6% and 17.4% in the chemoradiotherapy arm and 25% and 11.4% in the chemotherapy arm, respectively.

### Efficacy: LRRFS, DMRFS, DFS, and OS

**Figure 2:**
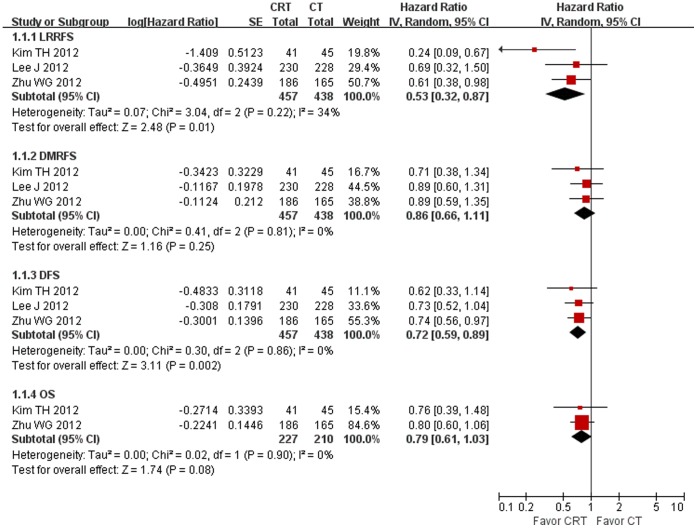
Forest plot of efficacy comparing chemoradiotherapy (CRT) with chemotherapy (CT).

895 randomized patients from 3 RCTs, 457 in the chemoradiotherapy group and 438 in the chemotherapy group, were included in the meta-analyses of LRRFS, DMRFS, and DFS. 437 randomized patients from 2 RCTs, 227 in the chemoradiotherapy group and 210 in the chemotherapy group were included in the meta-analysis of OS. The result of the test for heterogeneity of the treatment effects were not significant (P>0.10). Compared to chemotherapy, chemoradiotherapy significantly reduced the risk of locoregional recurrence and disease recurrence by 47% (P = 0.01) and 28% (P = 0.001), respectively. However, chemoradiotherapy didn’t significantly improve DMRFS (P = 0.26) and OS (P = 0.07). The detailed data was shown in Figure 2.

### Toxicities

**Figure 3:**
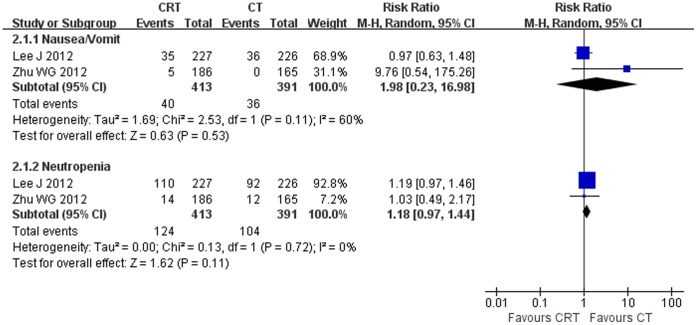
Forest plot of toxicities comparing chemoradiotherapy (CRT) with chemotherapy (CT).

Overall, toxicities in 3 selected RCTs were tolerable. The most common grade 3 or 4 adverse events were nausea, vomiting, hand and foot syndrome (only occurred in patients received capecitabine) and neutropenia. Pooled results suggested that there was no significant difference between two treatment approaches (Figure 3).

### Publication Bias Assessment

**Figure 4:**
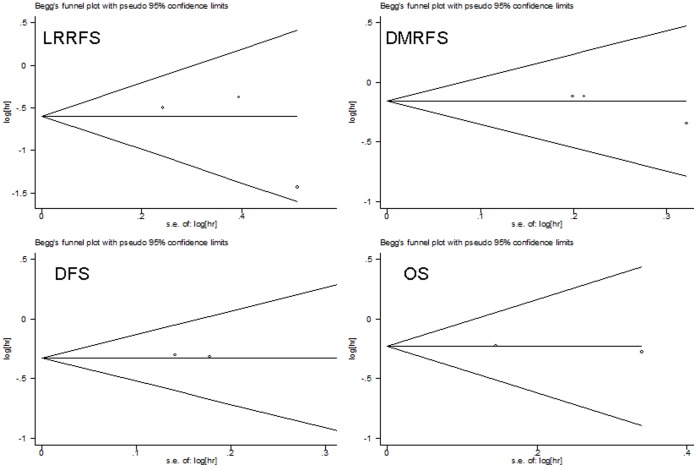
Begg’s funnel plot for publication bias.

Begg’s funnel plot and Egger’s test were performed to access the publication bias of literatures. The shapes of the funnel plots did not reveal any evidence of obvious asymmetry (Figure 4). Then, the Egger’s test was used to provide statistical evidence of funnel plot symmetry. The results still did not suggest any evidence of publication bias (Z = 1.04, P = 0.30 for LRRFS, DMRFS, and DFS, Z = 0, P = 1.00 for OS, respectively).

### Sensitivity Analyses

Sensitivity analyses were performed to evaluate whether the pooled estimates of LRRFS, DMRFS, DFS, and OS were different by exclusion of the highest weighted study and by omitting the trial that only included III/IV gastric cancer in each pooled analysis. Finally, the results were all consistent with the above outcomes.

## Discussion

Now, more and more surgeons accept D2 gastrectomy as required operation for patients with resectable gastric cancer. The optimal adjuvant therapy strategy is not well-defined in these patients. A Korean observational study suggests that postoperative chemoradiotherapy can prolong survival and decrease recurrence compared to observation [17]. In contrast, both single RCT and IPD-based meta-analysis suggest a survival benefit associated with postoperative chemotherapy [6], [7], [18]. Therefore, it is valuable to explore relative benefits of adjuvant therapies in this setting. Overall, our meta-analysis showed that postoperative chemoradiotherapy improved LRRFS and DFS, but didn’t improve DMRFS and OS compared with postoperative chemotherapy. To our knowledge, this was the first meta-analysis that compared postoperative chemoradiotherapy with postoperative chemotherapy in resected gastric cancer with D2 lymphadenectomy.

Although chemoradiotherapy improved LRRFS and DFS, good locoregional control didn’t transfer to OS benefit. Can we conclude that no survival benefit of chemoradiotherapy is principally a consequence of no DMRFS benefit? To answer this question, three facts should be emphasized. First, compared with D1 lymphadenectomy, D2 or more extended lymphadenectomy produces more reduction of local recurrence than that of distant metastasis. For example, in Dutch Gastric Cancer Group Trial, the locoregional recurrence is reduced by 13% (58% D1 group vs. 45% D2 group), which is higher than 7% of reduction in distant metastasis (48% D1 group vs. 41% D2 group). In a Taiwanese trial, the locoregional recurrence is reduced by 11% (30% D1 group vs. 19% D3 group), which is higher than 8% of reduction in distant metastasis (37% D1 group vs. 29% D3 group) [5], [19]. Second, predominant recurrence pattern associated with D2 lymphadenectomy was distant metastasis in Asian population [20]. In contrast, locoregional recurrence was more frequent than distant metastasis in West countries population who underwent D2 gastrectomy [21]. This viewpoint was also supported by results of RCTs selected in our present meta-analysis, in which distant metastasis rate is higher than local recurrence rate for Asian population (22.5%–43.3% vs. 6.6%–23%). At last, whatever type of lymphadenectomy is performed, postoperative chemoradiotherapy doesn’t reduce distant metastasis even compared with observation [3], [4], [17]. Taken together, bad DMRFS might offset LRRFS benefit from chemoradiotherapy for Asian patients underwent D2 gastrectomy.

To date, the reason that distant metastasis rate is higher than local recurrence rate for Asian population with gastric cancer after D2 lymphadenectomy is not very clear. A meta-analysis showed that there was a high percentage of diffuse-type histology gastric cancer in Asian population, which accounted for 50% at least [22]. Diffuse gastric cancer is prone to early metastasis, and for whom chemoradiotherapy does not appear to confer a benefit [23]. In present meta-analysis, patients were from Asian countries and diffuse-type gastric cancer accounted for more than 50% in two trials [8], [9]. We can't exclude that more diffuse-type gastric cancer selected in the two original trials was the main reason for higher rate of distant metastasis than that of locoregional recurrence. As commented by Brooks, if the finding of decreased efficacy of chemoradiotherapy in diffuse histology is confirmed, future trials may consider exploring different adjuvant approaches based on histology [23].

Our meta-analysis didn’t show that reduction of locoregional recurrence could transfer to OS benefit by adding radiation to postoperative chemotherapy in non-selected population. How is the result if we focused on subgroup of patients with pathologic lymph node metastasis at the time of surgery? A retrospective study shows that adjuvant chemoradiotherapy is associated with a significant improvement in survival for subgroup of patients with node-positive gastric cancer treated with D2 lymphadenectomy [24]. Subgroup analysis of ARTIST trial also shows that patients randomly assigned to the chemoradiotherapy arm experienced superior DFS when compared with those who received chemotherapy alone [8]. In contrast, in another Korean RCT which almost included patients purely with pathologic lymph node metastasis, intent-to-treat analysis doesn’t show that addition of radiation therapy to chemotherapy significantly improves DFS or OS [9]. Due to the inconsistent result, we couldn’t get a definite conclusion on the benefit of adjuvant chemoradiotherapy for patients with pathologic lymph node metastasis. We hoped that the ongoing ARTIST-II trial will give us a clear answer.

The main grade 3 or 4 toxicities were nausea/vomit and neutropenia irrespective of chemoradiotherapy or chemotherapy. Overall, meta-analysis didn’t find any difference in toxicities between two treatment approaches.

Although this meta-analysis was based on high-quality RCTs and was properly conducted, there are some typical limitations in our study. One major limitation is the number of trials is quite small and that possibly could not unveil the real situation, but the sample size of patients is amounted to 895. Another, all of the data was extracted from abstracted data (AD) instead of individual patient data (IPD), which would be less powerful to confirm our findings. However, a correlation analysis shows AD meta-analysis is strongly correlated with IPD meta-analysis [25], indicating AD as a kind of acceptable and practical method of meta-analysis alternative for IPD. The third, characteristics of patients were similar among selected trials, except for tumor stage. Stage I B-IV included in two selected trials [8], [10], in contrast with only stage III/IV gastric cancer was included in Kim et al’s trial [9]. However, the result of meta-analysis was not materially altered after omitting this trial (sensitivity analyses). In addition, the possible existence of unpublished studies should be aware of, which could lead to potential publication bias. However, no such bias was found by statistical methods. In general, regarding these limitations mentioned above, we should interpret the results with adequate caution.

In a summary, postoperative chemoradiotherapy might have no survival advantage over postoperative chemotherapy for non-selected Asian population with curable gastric cancer after D2 lymphadenectomy. However, diffuse-type histology and positive lymph node disease might have an important impact on patients benefit from different adjuvant therapies. At the present, limited number of trials limited further subgroup analysis to confirm our speculation. Future trials may consider exploring different adjuvant approaches for patients after D2 gastrectomy based on histology and lymph node status.

## Supporting Information

Checklist S1 (DOC)
