# Correction: Postoperative Chemoradiotherapy versus Postoperative Chemotherapy for Completely Resected Gastric Cancer with D2 Lymphadenectomy: A Meta-Analysis

**DOI:** 10.1371/annotation/e783be87-404d-4780-9159-bfd1ade5c3a4

**Published:** 2013-08-01

**Authors:** Yuan-Yuan Huang, Qiong Yang, Si-Wei Zhou, Ying Wei, Yan-Xian Chen, De-Rong Xie, Bei Zhang

The author contributions provided in the HTML and PDF versions of the article contain author initials from a different paper. The following are the correct author contributions for this article:

Conceived and designed the experiments: BZ YYH QY. Performed the experiments: BZ YYH QY SWZ YW YXC DRX. Analyzed the data: YYH QY SWZ YW YXC DRX. Contributed reagents/materials/analysis tools: YYH QY SWZ YW YXC DRX. Wrote the paper: YYH QY.

