# Supplementary material for: Postoperative Chemoradiotherapy versus Postoperative Chemotherapy for Completely Resected Gastric Cancer with D2 Lymphadenectomy: A Meta-Analysis
Source: PLoS One. 2013 Jul 18;8(7):e68939. doi: 10.1371/journal.pone.0068939 (PMC3715514; doi:10.1371/journal.pone.0068939)
Supplement: Checklist S1 — (DOC) [file pone.0068939.s001.doc]

| **Section/topic** | **#** | **Checklist item** | **Reported on page #** |
| --- | --- | --- | --- |
| **TITLE** | | |  |
| Title | 1 | Postoperative chemoradiotherapy versus postoperative chemotherapy for completely resected gastric cancer with D2 lymphadenectomy: A Meta-analysis | 1 |
| **ABSTRACT** | | |  |
| Structured summary | 2 | Background: Both chemoradiotherapy and chemotherapy are used in postoperative adjuvant therapy for resected gastric cancer. However, it is controversial whether chemoradiotherapy or chemotherapy is the optimal strategy for patients with gastric cancer after D2 lymphadenectomy. The present meta-analysis aims to provide more evidence on the relative benefits of adjuvant therapies in this setting.  Methods: We conducted a systematic review of randomized controlled trials, extracted time-to-event data using Tierney methods (when not reported), and performed meta-analysis to obtain the relative hazards of adjuvant chemoradiotherapy to chemotherapy on efficacy and toxicities.  Results: A total of 895 patients from 3 randomized controlled trials were identified for this meta-analysis. All patients were from Asian countries. Our results showed that postoperative chemoradiotherapy significantly improved locoregional recurrence-free survival [LRRFS: hazard ratio (HR) = 0.53, 95% CI = 0.32–0.87, p = 0.01] and disease-free survival (DFS: HR = 0.72, 95% CI = 0.59–0.89, p = 0.002); however, the improvement of distant metastasis recurrence-free survival (DMRFS: HR = 0.86; 95% CI = 0.66–1.11, p = 0.25) and overall survival (OS: HR = 0.79, 95% CI = 0.61–1.03, p = 0.08) were non-significant. The main grade 3 or 4 toxicities were equivalent between the two groups.  Conclusion: In non-selected Asian patients with resected gastric cancer who underwent D2 lymphadenectomy, postoperative chemoradiotherapy improved LRRFS and DFS but might not improve OS compared to postoperative chemotherapy. | 2 |
| **INTRODUCTION** | | |  |
| Rationale | 3 | To our knowledge, there are three phase Ⅲ randomized controlled trials (RCTs) which directly compared postoperative chemoradiotherapy with postoperative chemotherapy for patients with gastric cancer after D2 curative gastrectomy. Two in three RCTs do not find any differences in overall survival (OS) and disease-free survival (DFS) between the two approaches. One RCT suggests that chemoradiotherapy improves DFS compared with chemotherapy. In a summary, there was no consensus on whether chemoradiotherapy or chemotherapy should be the optimal adjuvant therapy for resected gastric cancer. | 4 |
| Objectives | 4 | Present meta-analysis aims to assess efficacy and toxicity on comparing chemoradiotherapy with chemotherapy in patients with resected gastric cancer after D2 lymphadenectomy. | 4 |
| **METHODS** | | |  |
| Protocol and registration | 5 | No protocol and registration. |  |
| Eligibility criteria | 6 | (1) patients with resected gastric cancer underwent D2 lymphadenectomy, (2) interventions and comparisons: chemoradiotherapy vs. chemotherapy in the setting of adjuvant therapy, (3) randomized controlled trials. | 5 |
| Information sources | 7 | Cochrane Central Register of Controlled Trials, PubMed, EMBASE, ISI Web of Knowledge, Chinese biomedical literature service system (SinoMed), ASCO abstracts, and ESMO abstracts were searched from the first available year to October 31, 2012, as well as hand searching of the references of identiﬁed articles and relevant meta-analysis. | 5 |
| Search | 8 | PubMed search strategy: ((((((chemoradiation OR chemoradiotherapy)) OR ("combined modality therapy"[Mesh]))) AND (((chemotherapy)) OR ("chemotherapy, adjuvant"[MeSH]))) AND ((((digest* OR gastr* OR gut OR epigastr* OR stomach*)) AND (((carcin* OR cancer* OR neoplas* OR tumour* OR tumor* OR cyst* OR adenocarcin* OR malig*)) OR ("adenocarcinoma"[Mesh]))) OR ("stomach neoplasms"[Mesh]))) AND (D2) | 5 |
| Study selection | 9 | Two investigators (Huang YY and Yang Q) screened each of the titles, abstracts, and full texts to determine inclusion independently. The results were compared and disagreements were resolved by consensus. | 6 |
| Data collection process | 10 | Information was carefully extracted from all included publications independently by two of the authors (Huang YY and Yang Q). Disagreement was resolved by consensus. If these two authors could not reach a consensus, another author (Zhang B) was consulted. | 6 |
| Data items | 11 | The following variables were extracted from each trial if available: total numbers of patients, age, sex, ECOG performance status, primary tumor site, Lauren classification, tumor stage, treatment regimens, endpoints, median follow-up time, Jadad scale score, and toxicities. | 6 |
| Risk of bias in individual studies | 12 | The quality of included studies was evaluated independently by two authors (Huang YY and Yang Q) of this article according to the Jadad Scale. A trial was regarded as high quality trial with high external and internal validities if it scored more than 3 points. | 5,6 |
| Summary measures | 13 | The principal summary measures are hazard ratio (HR) and 95% confidence interval (CI). | 6,7 |
| Synthesis of results | 14 | Crude HRs and RRs with 95% CIs were used to assess the survival benefit and risk of toxicities between chemoradiotherapy group and chemotherapy group, respectively. | 7 |

Page 1 of 2

| **Section/topic** | **#** | **Checklist item** | **Reported on page #** |
| --- | --- | --- | --- |
| Risk of bias across studies | 15 | Begg’s funnel plot and Egger’s test were performed to access the publication bias of literatures. | 7 |
| Additional analyses | 16 | Sensitivity analyses were performed to evaluate whether the pooled estimates of LRRFS, DMRFS, DFS, and OS were different by exclusion of the highest weighted study and by omitting the trial that only included Ⅲ/Ⅳ gastric cancer in each pooled analysis. | 9 |
| **RESULTS** | | |  |
| Study selection | 17 | 315 of studies screened, 7 assessed for eligibility, and 3 included in the review. Main reasons for exclusions included: non-RCT, non-blinded, no control arm, and not pure D2 lymphadenectomy. Flow diagram was shown in Figure 1. | 8 |
| Study characteristics | 18 | Table 1 shows the trials included in the meta-analysis and their main characteristics. | 8 |
| Risk of bias within studies | 19 | The Jadad scale scores were shown in Table 1. The Jadad scale scores were 3 points, indicating that the methodological quality was generally good. | 8 |
| Results of individual studies | 20 | The main results of individual studies were shown in Table 1 and Table 2, respectively | 8 |
| Synthesis of results | 21 | 895 randomized patients from 3 RCTs, 457 in the chemoradiotherapy group and 438 in the chemotherapy group were included in the meta-analyses of LRRFS, DMRFS, and DFS. 437 randomized patients from 2 RCTs, 227 in the chemoradiotherapy group and 210 in the chemotherapy group were included in the meta-analysis of OS. The result of the test for heterogeneity of the treatment effects were not significant (P>0.10). Compared to chemotherapy, chemoradiotherapy significantly reduced the risk of locoregional recurrence and disease recurrence by 47% (HR = 0.53, 95% CI = 0.32–0.87, P=0.01) and 28% (HR = 0.72, 95% CI = 0.59–0.89, P=0.001), respectively. However, chemoradiotherapy didn’t significantly improve DMRFS (HR = 0.86; 95% CI = 0.66–1.11, P=0.26) and OS (HR = 0.79, 95% CI = 0.61–1.03, P=0.07). | 8,9 |
| Risk of bias across studies | 22 | Begg’s funnel plot and Egger’s test did not suggest any evidence of publication bias. | 9 |
| Additional analysis | 23 | Sensitivity analyses were performed to evaluate whether the pooled estimates of LRRFS, DMRFS, DFS, and OS were different by exclusion of the highest weighted study and by omitting the trial that only included Ⅲ/Ⅳ gastric cancer in each pooled analysis. Finally, the results were all consistent with the above outcomes. | 9 |
| **DISCUSSION** | | |  |
| Summary of evidence | 24 | 3 randomized controlled trials were identified for this meta-analysis. All patients were from Asian countries. In Asian patients with resected gastric cancer who underwent D2 lymphadenectomy, postoperative chemoradiotherapy had no survival advantage over postoperative chemotherapy. | 10,13 |
| Limitations | 25 | One major limitation is the number of trials is quite small and that possibly could not unveil the real situation. Another, all of the data was extracted from abstracted data (AD) instead of individual patient data (IPD).The third, patient characteristics not consistent among selected trials, for example, tumor stage. | 12,13 |
| Conclusions | 26 | Postoperative chemoradiotherapy might have no survival advantage over postoperative chemotherapy for non-selected Asian population with curable gastric cancer after D2 lymphadenectomy. Future trials may consider exploring different adjuvant approaches for patients after D2 gastrectomy based on histology and lymph node status. | 13 |
| **FUNDING** | | |  |
| Funding | 27 | No fundings. |  |

*From:*  Moher D, Liberati A, Tetzlaff J, Altman DG, The PRISMA Group (2009). Preferred Reporting Items for Systematic Reviews and Meta-Analyses: The PRISMA Statement. PLoS Med 6(6): e1000097. doi:10.1371/journal.pmed1000097

For more information, visit: **www.prisma-statement.org**.

Page 2 of 2
